# Eight Weeks without Food

**Published:** 1891-08

**Authors:** 


### EIGHT WEEKS WITHOUT FOOD.

Little Lizzie Meadows has lain speechless, motionless, and without food for fifty-six days. The other day a reporter visited the Meadows' home in Walnut Park, a half deserted suburb of Independence. In a darkened room lay the girl, pallid as a corpse and as motionless, save for the quick drawn breath through her parted lips. Her shoulders, arms, and hands were emaciated beyond belief, her face, though absolutely colorless, being much less affected. An occasional lifting of the eyelids was the only perceptible motion, though Mrs. Meadows says her daughter is now able to lift one arm slightly.

The child is starved to pitiful gauntness. He face is waxen pale, thin, and drawn with agony, her arms are wasted till nothing but the skin covers the bones, and her slender fingers are transparent and fragile. Of her condition she is fully conscious, though she is speechless and absolutely motionless save for the twitchings of the face and the half lifting of the eyebrows.

On December 29, Lizzie was twelve years old, and though slender and delicate, she was in excellent health. She attended school a few days after the Christmas holidays closed, but soon began to complain of strange fleeting pains, first in the head, then about the chest, then in her finger tips. She was taken from school and was soon very ill—the physician who came said it was spinal meningitis.

The girl's condition grew serious. She lost the power of speech, of motion, and on March 21, she partook of the last morsel of food that she has tasted in eight weeks. Her parents and attending physicians tempted her with every conceivable dainty, but she was compelled to reject them all. Occasionally the motionless sufferer would take a few sips of lemonade or of water, and this, with an occasional draught of soda, her parents say, was the only approach to nourishment she has had during her long trial. Cistern water she could not endure, being able to drink only that taken from a neighboring spring.

Daily the doctors who puzzled themselves in vain over the strange case said, "The girl must die within twenty-four hours," and her patient mother, who has scarcely left her side, thought every feeble breath would be the last. But the girl, though fading daily, until but a skeleton remains, lived on. The physicians who came confessed themselvest at fault; no such case had ever before come under their observation. At times the girl's tongue became black, coated, hard and as dry her mother says, "as a cob." These coatings, however, were removed, their presence apparently affecting the sufferer little.

Through all these weeks the child's mind remained unaffected. She knew of her sufferings and was able, by changes of countenance, to answer questions. An offer of assistance which displeased her or a question to which she wished to answer "no," brought a frown ; a question which pleased her was answered by a pitiful ghost of a smile. Sometimes the mother, seeing that the child desired something, would put to her a dozen questions, all being answered by frowns ; then the little one would struggle hard to speak until she gasped for breath. Frequently she would weep in distress at her inability to make herself understood.

The doctors of Independence came and exhausted their skill in unavailing efforts to restore health to the child until last week, when one determined to apply a galvanic battery. The battery was used charily, a very feeble current being transmitted to the ehild through the hands of her brother and the physician, who each held a pole of the battery, lest a too strong shock might drive the life from the feeble body. There was at first no perceptible effect from this treatment, which was repeated daily, but at present it promises to release the girl from her living death.

Saturday morning the mother, as has been her wont, was breakfasting at her child's bedside, vigilantly watching the patient. The girl by her expectant countenance and parted lips manifested a desire for something.

"Do you want some breakfast, Lizzie?" asked the mother.

The child smiled.

Carefully the mother gave her a bit of buttered bread and an egg, which were swallowed with avidity, the little one mutely pleading for more. Three times again during the day did Mrs. Meadows and the physician give the child food in minute quantities, the morsels being eaten with no apparent evil effects. She was also given four meals Sunday, Monday and yesterday, and with daily applications of the galvanic battery the faster is beginning to exhibit signs of life.—*Ex.*

---

### THE ACT OF BREATHING.

In each respiration an adult inhales one pint of air. A healthy man respires 16 to 20 times a minute, or 20,000 times a day; a child 25 or 35 times a minute. While standing, the adult respiration is 22 times per minute; while lying down, 13. The superficial surface of the lungs, *i. e.*, of their alveolar space, is 200 square yards. The amount of air respired every 24 hours is about 10,000 quarts. The amount of oxygen absorbed in 24 hours is 500 litres (about 744 grams). The amount of carbonic acid expired in the same time is 400 litres (911.5 grams).

Two-thirds of the oxygen absorbed in 24 hours is absorbed during the night hours, from 6 p. m. to 6 a. m.; three-fifths of the total is thrown off during the day. The pulmonary surface gives off 150 grams of water daily in the state of vapor. An adult must have at least 360 litres of air in an hour. The heart sends 800 quarts of blood through the lungs every hour, or about 5,000 daily. The duration of inspiration is five-twelfths of expiration, seven-twelfths of the whole respiratory act. During sleep inspiration occupies ten-twelfths of the respiratory period.

### THE HUMAN EAR.

The human ear is an organ the true inwardness of which the physicians have never been able to get at. They can examine the interior of the eye with ease by throwing into its dark chamber a ray of light reflected from a little mirror, and of late they have found it possible even to see the gray matter of the brain by looking through the little canal by which the optic nerve enters. The cavity behind the nose they inspect with the aid of a light placed far back in the mouth. They have no difficulty in seeing into the stomach by an electric apparatus; the intestines likewise are readily enough investigated, and the bladder also. But the ear, as to its internal arrangements, is unapproachable. It is impossible to dissect it satisfactorily after death, for the reason that the parts collapse at once when the vital spark leaves the body.—*Ex.*

